# High serum levels of soluble CD40-L in patients with undifferentiated nasopharyngeal carcinoma: pathogenic and clinical relevance

**DOI:** 10.1186/1750-9378-2-5

**Published:** 2007-03-01

**Authors:** Laura Caggiari, Massimo Guidoboni, Emanuela Vaccher, Luigi Barzan, Giovanni Franchin, Annunziata Gloghini, Debora Martorelli, Paola Zancai, Maria Teresa Bortolin, Mario Mazzucato, Diego Serraino, Antonino Carbone, Paolo De Paoli, Riccardo Dolcetti

**Affiliations:** 1 Dept. of Pre-Clinical and Epidemiological Research, Centro di Riferimento Oncologico, IRCCS – National Cancer Institute, Aviano (PN), Italy; 2 Dept. of Medical Oncology, Centro di Riferimento Oncologico, IRCCS – National Cancer Institute, Aviano (PN), Italy; 3 Head and Neck Department, Azienda Ospedaliera, Pordenone, Italy; 4 Dept. of Radiotherapy, Centro di Riferimento Oncologico, IRCCS – National Cancer Institute, Aviano (PN), Italy; 5 Dept. of Pathology, Diagnostic Immunohistochemistry and Molecular Pathology Unit, Centro di Riferimento Oncologico, IRCCS – National Cancer Institute, Aviano (PN), Italy; 6 Blood Bank, Centro di Riferimento Oncologico, IRCCS – National Cancer Institute, Aviano (PN), Italy; 7 Dept. of Pathology, Istituto Nazionale Tumori, Milan, Italy; 8 Microbiology Unit, Centro di Riferimento Oncologico, IRCCS – National Cancer Institute, Aviano (PN), Italy; 9 Immunovirology and Biotherapy Unit, Centro di Riferimento Oncologico, National Cancer Institute, Via Franco Gallini 2, 33081, Aviano (PN), Italy

## Abstract

**Background:**

Engagement of CD40 promotes survival of undifferentiated nasopharyngeal carcinoma (UNPC) cells and similar effects are induced by the EBV oncoprotein LMP-1 that is expressed in a fraction of cases. Considering that CD40 may be activated also by the soluble isoform of CD40L (sCD40L), we investigated the serum levels of sCD40L in a series of 61 UNPC patients from Italy, a non-endemic area for this disease.

**Results:**

At diagnosis, serum samples of UNPC patients contained significantly higher levels of sCD40L than age-matched healthy controls (p < 0.001). High levels of sCD40L (i.e., >18 ng/ml) were more frequently found in patients <40 years of age (p = 0.03) and with distant metastases at presentation (p = 0.03). Serum levels of sCD40L were inversely associated with the expression of the EBV oncoprotein LMP-1 (p = 0.03), which mimics a constitutively activated CD40. The amount of sCD40L decreased in a fraction of patients treated with local radiotherapy alone. Moreover, CD40L^+ ^lymphoid cells admixed to neoplastic UNPC cells were detected in cases with high serum levels of sCD40L, suggesting that sCD40L is probably produced within the tumor mass.

**Conclusion:**

sCD40L may contribute to CD40 activation in UNPC cells, particularly of LMP-1-negative cases, further supporting the crucial role of CD40 signalling in the pathogenesis of UNPC. sCD40L levels may be useful to identify UNPC patients with occult distant metastases at presentation.

## Background

Undifferentiated nasopharyngeal carcinoma (UNPC) constitutes the most common epithelial malignancy occurring in the nasopharynx, and is characterized by peculiar epidemiologic and clinicopathologic features [1,2]. UNPC has a high incidence in Southern China and Southeast Asia, whereas it is a rare disease in Europe and North America. Moreover, compared to other head and neck carcinomas, UNPC shows higher invasive and metastatic potential, being frequently diagnosed only after dissemination [3-5]. The presence of a highly cellular lymphoid stroma, admixed with neoplastic cells, represents a peculiar morphologic feature of UNPC. The majority of infiltrating cells is constituted by non-neoplastic T lymphocytes, but other reactive elements such as macrophages, plasma cells, eosinophils, and neutrophils, are also present in varying proportions [6,7]. Although the role of lymphoid stroma in UNPC is still poorly defined, immunophenotypic analyses have demonstrated that infiltrating T lymphocytes and tumor cells express several immune regulatory receptor/ligand pairs [8,9], suggesting that biologically relevant interactions between the two cell components probably occur *in vivo*.

Multiple factors contribute to the pathogenesis of UNPC, including genetic predisposition, environmental factors, and Epstein-Barr virus (EBV) infection [1,2,10,11]. In particular, EBV genomes are detected in virtually all UNPC worldwide and are monoclonal, consistent with a causal role for the virus [1,2,10]. EBV infection in UNPC cells is characterized by the expression of a limited set of latent proteins, including EBNA-1, LMP-1, and LMP-2 [1,2,10]. The LMP-1 protein transforms B both lymphocytes and rodent fibroblasts ad may contribute to the invasive and metastatic potential of carcinoma cells [12,13]. Recent evidence indicates that LMP-1 functions as a constitutively active tumor necrosis factor (TNF) receptor being able to recruit cellular signal transducing molecules in the C-terminus of the protein, resulting in the activation of several signaling pathways in a ligand-independent manner [14]. In particular, LMP-1 shares several functional properties with activated CD40, a member of the TNF receptor superfamily, being able, in certain instances, to substitute for CD40 *in vivo *[15,16].

CD40 binds to a ligand (CD40L) which is an ~35 kDa transmembrane protein expressed on activated T cells, mast cells, basophilis, eosinophils, and activated platelets [17,18]. The interaction between CD40 and CD40L is crucial for B-cell activation, antibody production, isotype switching and cell-mediated immune responses [17,19,20]. Moreover, the CD40 pathway is also involved in regulating the growth and survival of normal and transformed epithelial cells [21,22]. In particular, CD40 triggering on UNPC cells may directly convey survival signals, being able to prevent Fas-mediated apoptosis [23]. Like other members of the TNF family, CD40L exists also as a soluble isoform (sCD40L) produced by enzymatic cleavage at a metalloproteinase-sensitive site in the membrane-proximal region of the extracellular domain of the molecule [24,25]. sCD40L is biologically active and can replace, at least *in vitro*, the normal T-cell-derived CD40L signal to CD40-bearing cells [24,25]. sCD40L can be detected in the serum of healthy individuals and elevated levels have been reported in patients with autoimmune diseases [26-28], hepatitis B virus infection [26], lymphoproliferative disorders [29], and lung cancer [30]. Nevertheless, no information is currently available on the presence and amount of sCD40L in the serum of UNPC patients.

We therefore sought to determine the serum levels of sCD40L in a series of UNPC patients from Italy, a non-endemic area for this disease. The study was undertaken with the aim to identify potential associations between circulating levels of sCD40L and the virologic and clinico-pathologic parameters of UNPC at diagnosis, and to define the predictive and prognostic value of serum sCD40L.

## Results

### UNPC patients have higher serum levels of sCD40L than healthy donors

**Figure 1:**
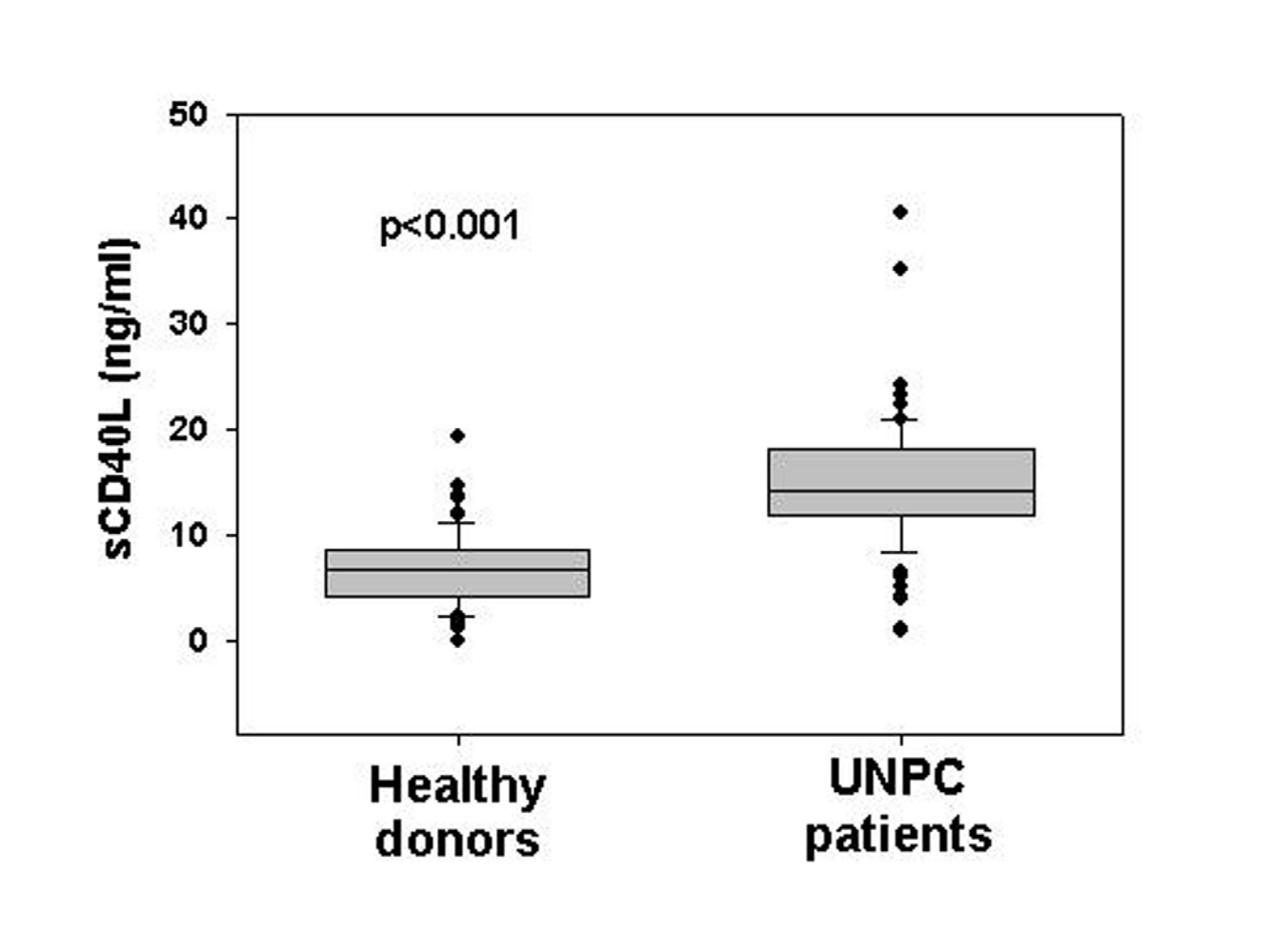
UNPC patients (n = 61) show increased levels of serum sCD40L compared to healthy donors (n = 71) matched for sex and age. The difference between the two groups was statistically significant (p < 0.001).

The individual and tumor-related characteristics of UNPC patients investigated are described in Table 1. Mean serum levels of sCD40L were significantly higher (approximately 3-fold) in UNPC patients than in healthy donors (15.2 ± 6.4 *vs*. 6.3 ± 3.6 ng/ml; p < 0.001) (Figure 1). Table 2 shows the results of the comparison of the sCD40L levels with regard to demographic and presenting clinical features of UNPC patients. Patients with sCD40L levels of in the highest category (>18 ng/ml) were more frequently <40 years old (8/16, 50% *vs*. 12/45, 26.7%; p = 0.03) (Table 2). No correlation was found between sCD40L levels and T stage or regional lymph node involvement (Table 2), whereas a significantly higher proportion of UNPC patients with distant metastases at presentation carried high amounts (>18 ng/ml) of sCD40L (6/8, 75% *vs*. 13/51, 25.5%; p = 0.03). Forty-one (69.5%) of the 59 UNPC patients investigated showed detectable amounts of EBV DNA in the serum (median: 16502 copies/ml; range 128–162.756 copies/ml), whereas 18 (30.5%) were negative. No association was found between serum sCD40L levels and EBV DNA load.

**Table 1:** Distribution of 61 patients with undifferentiated nasopharyngeal carcinoma (UNPC) according to selected characteristics. North-East Italy, 1994–2003.

**Characteristics**	**UNPC patients**
**Age **(years)	
Range	14–78
Median	49.6
**Sex**	N. (%)
Female	17 (27.9)
Male	44 (72.1)
**T stage**	
T1	13 (21.7)
T2	27 (45.0)
T3	11 (18.3)
T4	9 (15.0)
**N stage**	
N0	13 (22.1)
N1	23 (39.0)
N2	12 (20.3)
N3	11 (18.6)
**M stage**	
M0	51 (86.4)
M1	8 (13.6)
**Stage**	
I-II	20 (32.8)
III-IV	32 (52.5)
**Levels of sCD40L**	
High	20 (32.8)
Medium-low	41 (67.2)

**Table 2:** Comparison of patients with undifferentiated nasopharyngeal carcinoma according to sCD40L levels and personal characteristics and presenting clinical features. North-East Italy, 1994–2003.

**Characteristic^§^**	**Levels of sCD40L**				
	**High**		**Medium-Low**		**p**

	No.	(%)	No.	%	

**Age:**					
<= 39	8	(40.0)	8	(20.0)	
40–54	9	(45.0)	14	(35.0)	
>= 55	3	(15.0)	18	(45.0)	0.02
**Sex:**					
Men	16	(80.0)	28	(68.3)	
Women	4	(20.0)	13	(31.7)	0.51
**T:**					
1	4	(20.0)	9	(23.1)	
2A-2B	8	(40.0)	18	(46.2)	
3–4	8	(40.0)	12	(30.8)	0.78
**N:**					
0	2	(10.0)	11	(28.9)	
1	10	(50.0)	12	(31.6)	
2–3	8	(40.0)	15	(39.5)	0.36
**M:**					
0	13	(68.4)	38	(95.0)	
1	6	(31.6)	2	(5.0)	0.01
**Stage:**					
I-II	6	(31.6)	14	(42.4)	
III-IVa-IVb	7	(36.8)	17	(51.5)	
IVc	6	(31.6)	2	(6.1)	0.11
**LMP-1 expression**					
Negative cases	23	(95.8)	19	(70.4)	
Positive cases	1	(4.2)	8	(29.6)	0.03

^§^In some items, the sum does not add up to the total because of missing values

### Serum levels of sCD40L were inversely associated with LMP-1 expression by tumor cells

**Figure 2:**
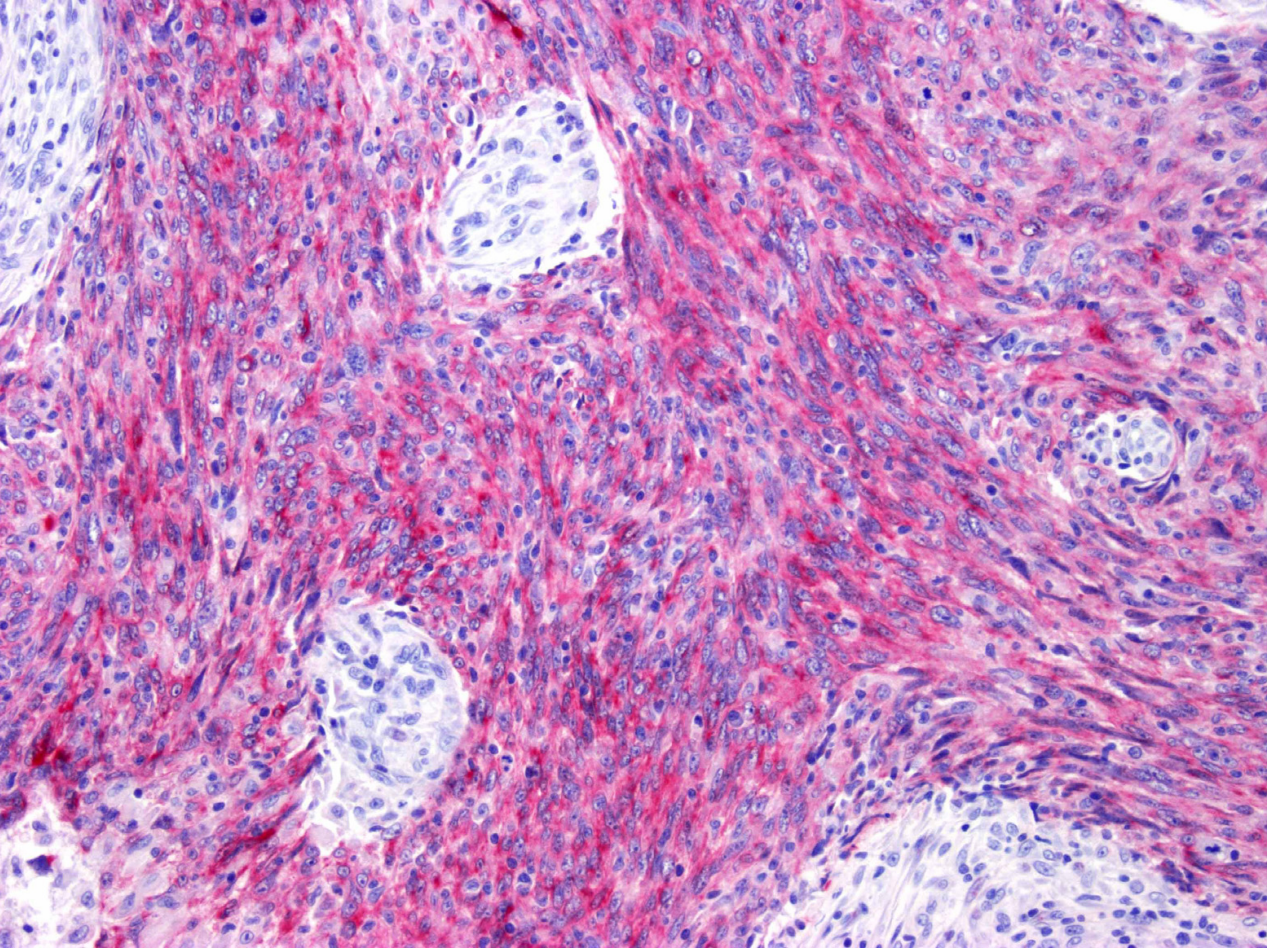
Detection of LMP-1 expression by immunohistochemistry (red) in UNPC tumor cells. Alkaline phosphatase anti-alkaline phosphatase, original magnification ×250.

Expression of LMP-1 was detected by immunohistochemistry in the large majority of tumor cells of 9 of 51 cases (17.6%) (Figure 2). Serum sCD40L levels were inversely associated with LMP-1 expression, as shown by the more frequent detection of high levels of sCD40L in LMP-1-negative cases (23/42, 54.8%), as compared to LMP-1-positive UNPC patients (1/9, 11.1%; p = 0.03) (Table 2).

### Serum sCD40L was not associated with the generation of active thrombin

sCD40L may be released by platelets activated as a consequence of thrombin generation [18,31]. Therefore, the pro-thrombin F1+2 fragment was analyzed as a coagulation activation marker because it provides an accurate estimate of the generation of active thrombin. Plasma samples from UNPC patients (n = 32) showed slightly higher levels of F1+2 compared with healthy donors (n = 30), although the mean levels detected in both groups were within the range of normality indicated by the manufacturer (0.91 ± 0.45 *vs*. 0.62 ± 0.26 nmol/L, not significant). Moreover, no correlation was found between the plasma F1+2 levels and the amount of serum sCD40L in the UNPC patients investigated for both analyses.

### Detection of CD40L expression on circulating T lymphocytes from UNPC patients

**Figure 3:**
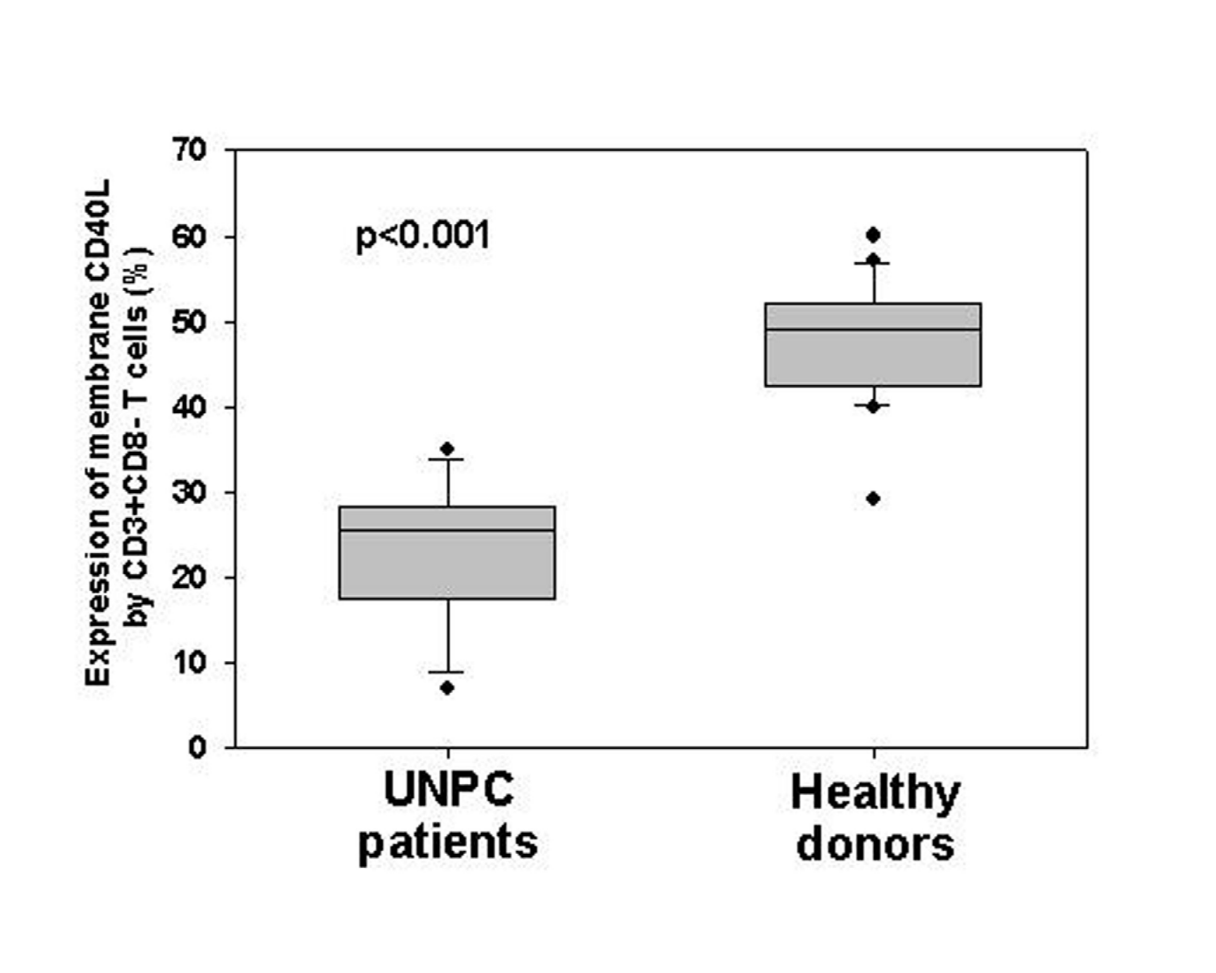
Membrane CD40L expression by stimulated CD3+CD8- circulating T lymphocytes in UNPC patients and healthy donors. The analysis was carried out using a two-color flow cytometric assay coupled with a previously described gating strategy [42]. The percentage of stimulated CD3+CD8- expressing membrane CD40L is indicated on the *y *axis. A significantly lower percentage of CD3+CD8- T cells from UNPC patients up-regulated CD40L upon stimulation as compared to controls (p < 0.001). The difference remained statistically significant also after correction for the number of CD4+ cells.

Membrane CD40L expression was investigated in circulating T cells from 9 UNPC patients and 16 healthy donors. Un-stimulated CD3+ T cells in both groups failed to express CD40L. After *in vitro *stimulation with PMA and ionomycin for 5 hours, CD3+CD8- cells from both UNPC patients and healthy controls displayed increased CD40L expression, whereas only a negligible number of CD3+CD8+ cells up-regulated CD40L (usually <2%). Notably, a significantly lower percentage of CD3+CD8- T cells from UNPC patients up-regulated CD40L upon stimulation as compared to controls (22.6 ± 9.1 *vs*. 47.4 ± 7.6; p < 0.001) (Figure 3). The difference remained statistically significant also after correction for the number of CD4+ cells (not shown).

### Expression of CD40L by tumor infiltrating lymphocytes

**Figure 4:**
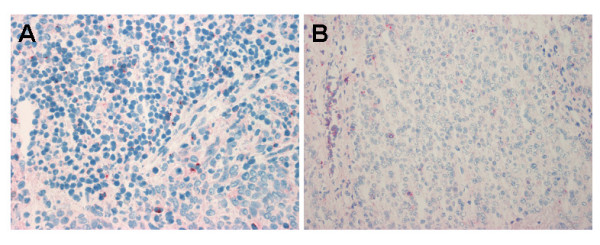
Immunohistochemical detection (on frozen tissue sections) of CD40L+ cells within UNPC microenvironment. CD40L positivity is manifested as dot-like staining on isolated small lymphocytes. **A**. The figure shows a high content of CD40L+ lymphocytes, either intermingled with tumor cells or located around UNPC tumor nests, in a case (female, 37 years old) with high serum levels of sCD40L. **B**. The figure shows few CD40L+ lymphocytes in a case (male, 34 years old) with a low content of sCD40L. Original magnification ×250.

Preliminary experiments carried out in frozen tissue sections from reactive lymph nodes, demonstrated that the expression of CD154/CD40L was restricted to small lymphocytes, confirming previous studies [32,33]. These cells displayed a prominent dot-like pattern or punctate para-nuclear staining, whereas a small number of positive cells exhibited a membrane staining pattern. The same analysis carried out in UNPC biopsies showed that tumor cells were CD40L-negative in all cases. Conversely, a variable number (1–10%) of non-neoplastic lymphoid cells in areas involved by UNPC were CD40L+. CD40L+ lymphocytes were intermingled with tumor cells and/or located around UNPC tumor nests. In all 5 cases, CD40L expression was correlated with the serum levels of sCD40L. The three cases with more than 5% CD40L+ infiltrating lymphocytes (Figure 4A) had higher levels of sCD40L. Conversely, two cases with a lower content of CD40L+ lymphocytes (<5%) (Figure 4B) were those with lower sCD40L levels.

### Treatment response and survival

All but seven patients completed the treatment, CR rate occurred in 82% of evaluable patients. After a median follow-up of 37.6 months (range 2.5–154), 16% and 11% of patients developed loco-regional and distant relapse, respectively. Distribution of treatment modalities and CR rate were comparable between patients with high (> 18 ng/ml) and medium-low (≤18 ng/ml) levels of serum sCD40L. Patients with higher sCD40L levels developed more frequently distant failure, whereas patients with medium-low levels developed loco-regional failure, but the difference in the pattern of relapse was of borderline significance (p = 0.07) (Table 2). Patients treated with RT alone had a marked decrease in serum sCD40L levels, but the average difference (3.9 ng/ml) between levels before and after RT was of borderline statistical significance (p = 0.07). The 5-year DFS and OS rates were 81% and 62%, respectively. There was no significant association between the sCD40L levels and outcome (Table 3). Patients with medium-low sCD40L levels had 5-year DFS and OS rates of 83% (95% CI: 68.5%–96.4%) and 62% (95% CI: 46.4%–70.2%), respectively, whereas patients with higher sCD40L levels had a 5-year DFS and OS of 80% (95% CI: 60%–100%) and 59% (95% CI: 35.3%–83.5%).

**Table 3:** Comparison of patients with undifferentiated nasopharyngeal carcinoma according to sCD40L levels and treatment-related factors. North-East Italy, 1994–2003.

**Characteristic^§^**	**Levels of sCD40L**				
	**High**		**Medium-Low**		**p**

Therapy	No.	(%)	No.	(%)	

CT	3	(15.0)	7	(17.1)	
RT	6	(30.0)	10	(24.4)	
CT+RT	11	(55.0)	24	(58.5)	0.89
**Response:**					
Complete remission	15	(83.3)	29	(80.6)	
Partial/Progression	3	(16.7)	7	(19.4)	0.80
**Recurrence**					
None	12	(80.0)	20	(69.0)	
Loco-regional	0	(0.0)	7	(24.1)	
Distance	3	(20.0)	2	(6.9)	0.07

^§^In some items, the sum does not add up to the total because of missing values.

## Discussion

The CD40-CD40L interaction plays a central role in the regulation of immune responses and mediates diverse biological responses that may contribute to the growth and survival of tumor cells. The relevance of CD40-dependent signaling in tumor development is also emphasized by the observation that the oncogenic properties of the EBV protein LMP-1 are due to its ability to hijack part of the CD40 cascade [14-16]. In the present study, we demonstrate that UNPC patients have significantly increased serum levels of sCD40L compared with healthy donors of similar sex and age. Interestingly, UNPC patients 40 years-old or younger carried higher amounts of serum sCD40L, an observation that further supports the hypothesis that UNPC of young people has distinctive epidemiologic and clinico-pathologic features [1,2]. In fact, while in endemic areas UNPC does not usually occur before 45 years of age [34], in North Africa this tumor also affects young people [1,2,7,35-37], representing 25% of all UNPC-affected patients [38]. Italian UNPC patients have similar epidemiologic features, as shown by a previous retrospective study [39] and by the finding that 28% of patients from the present series were 40 years-old or younger.

Several lines of evidence indicates that tumor cells may shed pro-coagulant factors that can be responsible for the generation of active thrombin [40]. This, in turn, may lead to platelet activation and the consequent release of various factors, including sCD40L [3,41]. In UNPC patients, however, the mean plasma levels of F1+2 were within the normal range, excluding thus an increased generation of active thrombin. These findings, together with the lack of correlation between the F1+2 levels and the amount of serum sCD40L, rule out that sCD40L is mainly released from platelets in UNPC patients. Moreover, the lack of membrane CD40L in circulating T cells also excludes that a systemic activation of the immune system may be responsible for enhanced release of sCD40L. Interestingly, upon activation, the percentage of CD40L+ T helper cells was significantly reduced in UNPC patients as compared to controls. Similar findings were also observed in HIV-infected children [42]. This effect is probably not due to the loss of distinct subpopulations of T helper lymphocytes, since Italian UNPC patients carry percentages and absolute numbers of CD4+ memory (CD45R0+), CD4+ naive (CD45RA+/CD62L+) and activated (HLA-DR+) CD4+ lymphocytes similar to those of healthy donors [[43], our unpublished results]. Our findings rather support the hypothesis of an underlying functional defect of T cell responses in these patients, as also suggested by previous reports [44,45]. In particular, some of us have recently demonstrated that circulating CD4+ T lymphocytes from Italian UNPC patients showed impaired IL-2 secretion and increased IL-10 production, consistently with a Th1/Th2 dysregulation [43]. Nevertheless, the nature and the mechanisms responsible for these functional abnormalities are not yet established and warrants further investigation.

Unlike what observed in circulating T cells, tumor infiltrating lymphocytes expressed membrane CD40L in all cases investigated, consistently with previous findings [8]. Besides strengthening the pathogenic relevance of the interactions occurring between UNPC cells and infiltrating lymphocytes, these results also suggest that sCD40L may be released within tumor microenvironment by activated T cells. This possibility is also supported by the finding that the amount of sCD40L in the serum may decrease in patients treated with local radiotherapy alone. Intriguingly, serum levels of sCD40L were inversely correlated with LMP-1 expression by tumor cells, suggesting that LMP-1-negative UNPC cells may require CD40 activation for growth and/or survival. These findings are in line with the known ability of LMP-1 to usurp part of CD40-dependent signalling and reinforce the notion that CD40 activation is of pathogenic relevance for UNPC [8,15,16,23].

Within tumor tissue, CD40 triggering by both CD40L+ lymphocytes and sCD40L may directly activate signalling pathways contributing to the transformed phenotype of UNPC cells, particularly in the LMP-1-negative cases. It is worth considering that CD40 activation may enhance the invasive potential of a variety of cells through the induction of matrix metalloproteinases [46-49], enzymes that efficiently degrade extracellular matrix proteins [50,51], favoring thus the metastatic process. Indeed, in our UNPC series, serum sCD40L levels were not correlated with T stage or regional lymph node involvement, ruling out any relationship with loco-regional tumor burden. Conversely, a significantly higher proportion of UNPC patients with distant metastases at presentation carried high levels of sCD40L, suggesting a possible contribution of this factor to an early hematogenous spreading of UNPC cells. The high levels of sCD40L detected in the serum of UNPC patients may also prevent apoptosis of UNPC cells circulating in the blood and favour their survival and/or growth after localization at distant sites. Moreover, considering that matrix metalloproteinases are involved in the generation of sCD40L [24,24], local production of these enzymes induced by CD40 triggering may in turn enhance the release of sCD40L.

While sCD40L levels may be useful to identify UNPC patients with occult distant metastasis at presentation, our study indicate that the amount of serum sCD40L does not provide a reliable estimate of tumor burden. This is in keeping with the hypothesis that sCD40L is not a product of tumor cells, being rather part of a still poorly defined anti-tumor immune response. On these grounds, additional studies are required to better understand the role played by sCD40L within the frames of the complex immune response mounted by UNPC patients against tumour cells. Moreover, no significant prognostic role on clinical outcome was found for serum sCD40L in our series. It should be considered, however, that 5-year DFS (81%) and OS rates (62%) of our patients compare favorably with the results of other UNPC series [52-54]. These data suggest that, if active treatment strategies are used, the prognostic impact of variables such as sCD40L may be measurable only in larger series. On these grounds, it would be relevant to include the evaluation of serum sCD40L among the parameters of possible prognostic value in studies enrolling high number of UNPC patients from both endemic and non-endemic areas.

## Conclusion

The results of the present study indicate that sCD40L may contribute to CD40 activation in UNPC cells, particularly among LMP-1-negative cases, further supporting the crucial role of CD40 signalling in the pathogenesis of UNPC. sCD40L levels may be useful to identify UNPC patients with occult metastases at presentation.

## Methods

### Patients characteristics

Sixty-one patients with histologically confirmed UNPC were enrolled between 1994–2003 (Table 1). The medical records were reviewed and all patients were restaged according to the 2002 edition of the UICC/TNM classification system. All patients received various treatment modalities, such as cisplatin-based combination chemotherapy (CDDP-CT) alone (10 cases), accelerated radiotherapy (RT) alone (16 cases) or combined treatment including neo-adjuvant CDDP-CT plus accelerated RT (25 cases) or CDDP-CT plus standard RT (10 cases). Complete response (CR) was defined as the complete disappearance of all cancer lesions for at least 4 weeks. Partial response (PR) was defined as 50% reduction of the sum of the products of the cross-sectional diameters of all known lesions for at least 4 weeks. No response (NR) was defined as <50% PR or progressive disease. Fifty-four (89%) patients were assessable for treatment response. Seven (11%) patients were not assessable because of early deaths due to co-morbidity (4 cases) or toxicity (3 cases). As control group, 71 healthy donors matched for sex and age (51 males, 20 females; mean age of 47.3 ± 11.3 ranging from 20 to 68 years) were also evaluated. Informed consent was obtained from each subject.

### Histopathological diagnosis and immunohistochemistry

Primary and metastatic UNPC were diagnosed according to histologic criteria provided by W.H.O. classification for nasopharyngeal carcinomas [7]. Cases with morphological characteristics of non-keratinizing undifferentiated carcinoma [7] were selected for the study. EBV infection was detected in all cases by EBER *in situ *hybridization performed as previously reported [55]. Immunohistochemistry for LMP-1 expression was carried out on paraffin-embedded tissue sections using the CS1-4 antibody (DakoCytomation, Glostrup, Denmark) and the APAAP method [56]. Immunohistochemical detection of CD40L was carried out on frozen sections obtained from 4 nasopharyngeal samples and 1 lymph node involved by UNPC by the APAAP technique [56] and the anti-CD154/CD40L 24–31 antibody (IgG1; Ancell Corporation, Bayport, MN). In each case, the percentage of CD40L+ lymphocytes was derived from the number of CD40L+ lymphocytes counted on a total of 100 lymphocytes evaluated. Negative control experiments were performed by incubating sections with irrelevant isotype-matched mouse Ig and by omitting the primary antibody.

### Sample collection and immunoassays

Serum samples were collected from 61 UNPC patients and 71 matched healthy donors. Plasma was also obtained from 32 UNPC patients and 30 donors using Na citrate 3.8% (1:9 v:v) as anticoagulant, immediately centrifuged at 1,500 × *g *at 4°C for 20 min, aliquoted, and stored at -80°C until analysis. Measurement of serum sCD40L levels was carried out using a commercially available enzyme-linked immunosorbent assay (ELISA) based on the sandwich principle, according to manufacturer's instructions (Immunokontact; sensitivity: 0.0095 ng/ml). Prothrombin fragment 1+2 (F1+2) levels were measured in plasma samples by a commercially available enzyme immunoassay (Enzygnost F1+2; Dade-Behring; reference range: 0.4–1.1 nmol/L). Measurements were done blinded. All samples were assayed in duplicate, and those showing values above the standard curve were re-tested with appropriate dilutions.

### EBV DNA load quantitation

Serum EBV DNA levels were measured by amplifying a 65-bp DNA fragment of the LMP-1 gene using a previously described real-time PCR assay [57]. The reaction was performed in a 25 μl volume containing 2× TaqMan Universal Master Mixture, 1.5 mM MgCl_2_, 99 pmol of each primer (forward: 5'-AAGGTCAAAGAACAAGGCCAAG-3', nucleotides 168,231–168,252; reverse: 5'-GCATCGGAGTCGGTGGG-3', nucleotides 168,188–168,204), 175 pmol of the fluorogenic probe (5'-6-FAM-AGGAGCGTGTCCCCGTGGAGG-TAMRA-3') and 5 μl of sample. Standard curves were generated using the diploid Namalwa cells, which stably harbours two integrated copies of the EBV genome. The method was linear over four orders of magnitude and sensitive to as few as 5 copies of template EBV DNA. To check for PCR inhibition, each sample was also co-amplified with a positive control (500 EBV DNA copies/reaction from Namalwa cells). Results were expressed as copies of EBV genomes/ml of serum.

### Immunophenotypic analyses

A two-color flow cytometric assay was used to study CD40L expression on resting and activated circulating T cells. Briefly, peripheral blood mononuclear cells (PBMCs) were isolated from citrated venous blood by centrifugation over Ficoll-Hypaque (1500 rpm, 30 min). Isolated PBMCs were washed three times with PBS and incubated at room temperature for 20 min with appropriate antibody combinations. Activation of T cells was carried out by incubating purified PBMCs (0.4 × 10^6 ^cells/ml) in RPMI 1640, 10% FCS in the presence of 20 ng/ml phorbol 12-myristate 13 acetate (PMA, Sigma, Milan), and 0.75 μg/ml ionomycin (Sigma) for 5 hr in 5% CO_2 _at 37°C. Cell were harvested, washed and labeled with CD40L-PE/CD3-FITC or CD40L-PE/CD8-FITC antibody combinations. CD40L expression on T helper lymphocytes was evaluated using a previously described gating algorithm [42] which included both lymphocytes (using forward scatter parameters) and CD3+CD8- T lymphocytes (using fluorescence parameters). This gating strategy allowed the evaluation of CD4+ lymphocytes (contained in the population of CD3+ CD8- T cells) and was necessary because the CD4 molecule itself is down-modulated after stimulation. Results are expressed as either the percentage of CD3+CD8- T lymphocytes expressing CD40L or the level of CD40L expressed per T helper cell (mean fluorescence intensity). Isotype-matched FITC- and PE-conjugated mouse IgG1 (Beckman-Coulter, Milan) were used as negative controls. After incubation, each sample was washed twice with PBS, re-suspended in PBS and immediately analyzed in an Epics Altra flow cytometer (Beckman-Coulter). A total of 10.000 cells were acquired and analyzed by Expo 32 software (Beckman-Coulter).

### Statistical analysis

sCD40L levels were categorized in two groups based on frequency distribution: medium-low, (≤18 ng/ml), or high (>18 ng/ml). The association between sCD40L levels and personal characteristics, clinical and biological parameters was tested by means of the chi-square test (chi-square test for trend, when appropriate) or the Student's T-test. The overall survival (OS) was computed from the date of UNPC diagnosis to death or to the last known date of examination, whereas the disease-free survival (DFS) was calculated (for CR patients only) from the date at starting therapy to date of relapse, death or the last known date of examination. Both the OS and the DFS were statistically assessed by means of the Kaplan-Meyer method.

## Competing interests

The author(s) declare that they have no competing interests.

## Authors' contributions

LC carried out the immunoassays, participated in the immunophenotypic analyses and drafted the manuscript. MG carried out the immunophenotypic assays and contributed to draft the manuscript. AG and AC were responsible for the histopathologic characterization and immunohistochemical findings and participated in drafting the manuscript. MM and DM carried out Prothrombin fragment 1+2 assays. PDP and MTB carried out the EBV DNA load quantitation. DS carried out the statistical analyses. EV, LB, and GF contributed to patient selection, collection and analysis of clinical data. DS participated in the design of the study and performed the statistical analysis. RD conceived of the study, and participated in its design and coordination and helped to draft the manuscript. All authors read and approved the final manuscript.
